# Dental caries and oral health practice among 12 year old school children from low socio-economic status background in Zimbabwe

**DOI:** 10.11604/pamj.2013.14.164.2399

**Published:** 2013-04-29

**Authors:** Brighton Tasara Mafuvadze, Lovemore Mahachi, Benford Mafuvadze

**Affiliations:** 1 Department of Dentistry, College of Health Sciences, University of Zimbabwe, Zimbabwe; 2 Department of Biomedical Sciences, University of Missouri, USA

**Keywords:** Dental caries, children, urban and rural area, Zimbabwe

## Abstract

**Introduction:**

Dental caries is one of the most prevalent chronic diseases affecting children in Sub-Saharan Africa. Previous studies show a higher prevalence of dental caries in children from low socio-economic status backgrounds. The purpose of this study was to determine the prevalence of dental caries among 12 year old children in urban and rural areas of Zimbabwe and establish preliminary baseline data.

**Methods:**

A descriptive cross-sectional study was conducted among 12 year old children at primary schools in Harare and Bikita district. A Pre-tested questionnaire was administered to elicit information from the participants on tooth cleaning, dietary habits and dental experience. Dental caries status was assessed using the DMFT index following World Health Organization (WHO) guidelines.

**Results:**

Our results showed a high prevalence of dental caries in both urban (59.5%) and rural (40.8%) children. The mean DMFT in urban and rural areas was 1.29 and 0.66, respectively. Furthermore, our data showed a general lack of knowledge on oral health issues by the participants.

**Conclusion:**

There is high prevalence of dental caries among 12 years old school children in both urban and rural areas of Zimbabwe. This calls for early preventive strategies and treatment services. We recommend incorporation of oral health education in the elementary school curricula.

## Introduction

Dental caries remains a major oral health disease affecting children world-wide [1]. While the prevalence and severity of dental caries in most industrialized countries have declined substantially in recent years, in developing countries like Sub-Saharan Africa the prevalence is predicted to increase [2]. This disparity between industrialized and developing countries has been attributed to preventive oral health care programs adopted by the former and changes in dietary habits coupled with inadequate exposure to fluorides in developing countries [2]. According to the World Health Organization (WHO) the prevalence of dental caries among school aged children is estimated to be as high as 90% in some countries [3]. For children in particular, poor oral health can have negative impacts on quality of life and academic performance at school [4]. Apart from causing chronic pain and discomfort, untreated dental caries can impact daily activities in terms of play, sleep, eating and school activity [5, 6].

Establishing baseline data on children dental caries and other oral health issues through regular national surveys is crucial for planning and development of intervention programs [1]. Unfortunately, most countries in Sub-Saharan Africa focus on high mortality diseases like HIV and AIDS, cancer, tuberculosis, diabetes and malaria and pay little attention to oral health issues. No study has investigated the prevalence of dental caries among school children in Zimbabwe in the last decade rendering it difficult to understand the status and pattern of oral health. To our knowledge, the last oral health survey targeting children less than 12 years old was done in Zimbabwe in 1985 by Chironga et al [7]. The other national survey done in 1995 covered adolescents and adults [8]. Both surveys, however, showed that the prevalence of dental caries in Zimbabwe was high across all age groups. The occurrence of dental caries has, however, been observed to widely vary from place to place and from time to time depending on socio-demographic characteristics such as family income [9]. When faced with hard economic challenges, it is likely that most people will give less priority to oral health issues. Since the last oral health survey, Zimbabwe experienced major economic challenges culminating in widespread poverty and high unemployment rates [10]. We hypothesized that the socio-economic situation that prevailed in Zimbabwe between 2000 and 2010 resulted in an increase in dental caries among school-going children. Previous studies show a higher prevalence of dental caries in children from low socio-economic status backgrounds [11]. Apart from the highlighted economic challenges, the introduction of multi-currency use in Zimbabwe in 2009 indirectly led to an increase in consumption of high sugar-containing products which are known to promote development of dental caries in children [12]. Due to unavailability of low denominations and coins for the major currencies currently in use such as the South African rand and the USA dollar, most people find it convenient to use easily available sugary products like sweets and chocolate during purchasing transactions as change. The frequent and high consumption of sugars is the major cause of dental caries in children [12].

The main focus of this study was to determine the prevalence of dental caries among twelve year old school children from low socio-economic status backgrounds in both urban and rural areas of Zimbabwe. Our results show a high prevalence of dental caries in both urban and rural children.

## Methods

**Study design:** This is a descriptive cross-sectional study designed to assess dental caries experience among 12 year old school children from low socio-economic status background attending public primary schools in Zimbabwe.

**Setting and study population:** For convenience purposes, participants in this study were drawn from two primary schools in urban (Harare, the capital city) and rural (Bikita district, located 400km,south of Harare) areas respectively. The study population consisted of 12 year old children attending public primary schools. We particularly focused on this age group as it is the WHO recommended index age [13].

**Sampling method:** For convenience purposes two public primary schools were selected in Harare (urban) and Bikita (rural area). The schools were selected to represent high and middle density urban and rural communities since our goal was to assess dental caries in children from a low socio-economic status background. At participating schools, all students with written consents were included in the study and examined. The sample size was estimated using Dobson's formula by applying an estimated dental caries prevalence of 15%, a design effect of 2, and a precision of 0.05 and was determined as 170 children.

**Data collection technique and tools:** A pre-tested questionnaire comprising information about the children's demographic background, oral health knowledge, attitudes and practices, dietary habits and dental services utilization history was administered and completed by participating children under supervision. Dental caries experience among the participants was assessed using the Decayed, Missing and Filled Teeth (DMFT) index, which is a measure of life time dental caries experience in permanent dentition. Clinical oral examination of the children was performed by one of the authors (BTM, a qualified practising dentist) following World Health Organization (WHO) diagnostic criteria [13]. All examinations were done in the classrooms with the children seated in an upright chair.

**Ethical consideration:** The study was reviewed and approved by the University of Zimbabwe Joint Ethics and Research Committee. In addition, permission was sought and granted by the Ministry of Education Sport, Arts and Culture. At participating schools, permission was sought from headmasters, teachers, and only children whose parents/guardians gave written consent were included in the study.

**Data analysis:** Collected data were entered, cleaned and stored using Epi-info-statistical package-7. Statistical analysis was done using the T-test, Chi-square and Wilcoxon test. Association between variables such as gender, socio-economic status and location on dental caries occurrence was determined. Significance level was set at p<0.05.

## Results

A total of 172 children, 79 (38 male and 41 female) from a middle-density urban area and 93 (36 male and 57 female) from a rural area school participated in this study.

### Dental caries status in participating children

Table 1 below shows the prevalence of dental caries among participants in this study. The prevalence of dental caries was significantly (p<0. 05) higher in children at the sampled urban school than those from a rural school. Our results showed a mean DMFT of 1. 29 for the urban school and 0. 66 for the rural school respectively. Similar to previous studies, most (96. 3%) of the teeth affected by dental caries were characterized and classified as decayed (Table 1). Interestingly, a majority of the students whose teeth were affected by caries remained untreated with only one participant from the urban school identified to have a filled tooth (Table 1). Gender differences were observed with respect to dental caries occurrence. While in the rural school, girls had a higher prevalence of dental caries than boys, in the urban school; boys had a higher prevalence than girls (Table 1). In terms of distribution, most teeth observed in this study to have dental cavities were lower jaw teeth and overall, the occlusal surface tended to be the most affected. On the other hand, first lower molars accounted for 44. 8% of the affected teeth.

**Table 1:** Dental caries experience in participating children from rural and urban schools

	Rural (N=93)		Urban (N=79)	
Gender	n (%) with caries	Index	n (%) with caries	Index
Male	12 (33.3)	0.47*	25 (65.8)	1.42
Female	26 (46.6)	0.77*	22 (53.7)	1.17
**DMFT component**	**Number of teeth**	**Index**	**Number of teeth**	**Index**
Decayed teeth	60	0.65*	97	1.22
Missing teeth	1	0.01	4	0.05
Filled teeth	0	0.00	1	0.01
DMFT	61	0.66*	102	1.29

DMFT: Decayed, missing and filled teeth; Index was calculated as the number of affected teeth divided by the total number of children

* Significantly different from corresponding urban group at p<0.05

### Dietary habits and oral health practices of participating children

Table 2 below shows the frequency at which children reported consumption of dietary products commonly associated with development of dental caries in this age group. Our results showed a higher consumption of high sugar-containing products like sweets, chocolate and soda drinks by urban children. Not surprising, a significant association between regular consumption of sugary products and a high DMFT was observed among urban children. As shown in Table 2, urban children who reported regular consumption of sweets and chocolates had significantly (p<0. 05) more dental caries than those who reported rare consumption of sugary products. Contrary to our hypothesis, rural children reported moderately low consumption of high sugar-containing dietary products suggesting that the observed high prevalence of caries in these children could be attributable to other factors. As shown in Table 2, nearly 70% of rural children reported no use of commercial fluoridated tooth paste +in contrast to 30% from the urban school. Our combined data showed that use of fluoride-containing toothpaste significantly reduced the risk of developing dental caries (Table 2). Although there were no major differences in teeth cleaning frequencies, a third of the children from the rural school reported use of chewing sticks while all urban children reported use of commercial tooth brushes. Confirmation of the reported oral practices was beyond the scope of this study, however, we observed an inverse relation between frequency of cleaning and occurrence of dental caries. As shown in Table 2, nearly all of the children who reported not cleaning their teeth regularly had dental caries at advanced stages. Interestingly, of those children who reported ever consulting a dentist the majority had dental caries.

**Table 2:** Dietary habits and oral practices among participating children and associated caries prevalence

	Rural (N=93)		Urban (N=79)	
Dietary habits	n	% with caries	n	% with caries
**Consumption of sweets**				
Everyday	0	0	13	53.8**
Several times a week	6	33.3	48	66.6**
Occasionally but not that often	87	41.4	18	50.0
**Consumption of chocolates**				
Everyday	0	0	2	50.0**
Several times a week	2	0	22	68.2**
Occasionally but not that often	91	41.8	55	58.2
**Consumption of soda drinks**				
Everyday	3	33.3	14	78.6**
Several times a week	14	42.8	44	58.8**
Occasionally but not that often	76	40.7	21	52.3
**Oral health practices**				
Frequency of tooth brushing				
Once/day	40	40.0	31	64.5**
Twice /day	18	38.9	41	56.1**
After every major meal	29	34.5	7	57.1**
Less often than daily	6	83.3	0	0
Tooth cleaning aids				
Toothbrush and toothpaste	25	24.0	53	50.9**
Toothbrush only	33	39.4	26	76.9**
‡Chewing stick	29	48.3	0	0
none	6	83.3	0	0
Consulted a dentist at least once	3	66.6	20	70.0

‡ Piece of stick from a tree that is flayed out at the end for brushing teeth.

** Significantly higher than corresponding rural group, P<0.05.

### Knowledge of oral health issues by participating children

We explored the participants’ knowledge and attitudes on causes of dental caries and preventative measures against the disease. Overall, urban children were more knowledgeable than rural children with no significant gender differences. Overall, the majority of children were knowledgeable of the potential role of sweets and other sugary products in the development of dental caries (Table 3). Interestingly, nearly 40% of rural children subscribed to a commonly held misperception that dental caries is caused by tooth worms. Overall, most children were knowledgeable and aware of preventive measures such regular cleaning of teeth, reduced consumption of sugary products and use of fluoridated tooth paste (Table 3). Most rural children reported receiving information on oral health either at home or at school. On the other hand, only 48% of urban children reported getting information at home and most reported getting minimal information at school. In agreement with previous studies, very few (5.8%) of the children reported ever getting information on oral health from a professional dentist.

**Table 3:** Knowledge of potential causes of dental caries and preventive measures

Causal factors	n (%) Correctly identified factor	
	Rural (N=93)	Urban (N=79)
High sugar-containing foods	77 (83)	72 (91)
Poor oral hygiene	66 (71)	55(70)
Bacteria	27 (29)	63 (80)
Tooth worm	58 (62)	72 (91)
Preventive measures		
Tooth brushing	66 (71)	55 (70)
Consuming less sugary products	76 (82)	72 (91)
Rinse mouth after a major meal	55 (59)	39 (49)
Consulting a dentist	81 (87)	66 (84)
Use of fluoridated toothpaste	76 (82)	50 (64)

## Discussion

Dental caries continue to be a major oral health concern for children in Sub-Saharan Africa. In this study we observed a higher prevalence of dental caries in both urban and rural children of similar age. Our study confirms previously reported trends in Sub-Saharan countries [14], which show higher prevalence of dental caries among urban children compared to their rural counterparts. This disparity in dental occurrence between urban and rural children has been partially attributed to increased access and consumption of high sugar-containing foods and beverages in urban areas [14]. While most people in rural areas in Zimbabwe cannot afford and perceive these sugary-products as non-beneficial, affording them is often considered a symbol of higher socio-economic status. In contrast to our prediction, the consumption of sugary products in rural areas is still moderately low. Given the low consumption of sugary products reported by rural children in this study, the higher prevalence of dental caries is potentially due to other factors. Economic challenges tend to have severe impact on rural people since they have limited financial resources. This is quite evident from this study as shown by limited availability of basic oral hygiene products such as fluoride-containing tooth pastes and the widespread use of wooden sticks for cleaning teeth reported by rural children.

Despite reporting high use of commercial fluoridated toothpaste and frequent teeth cleaning, the prevalence of dental caries (59.9%) observed among urban children in this study is significantly higher than what was reported recently in similar studies in Sudan [15], Uganda [16], Tanzania [17], and Kenya [6]. We hypothesize that this might be a direct reflection of socio-economic situation in Zimbabwe and partially a consequence of the increased consumption of high sugar-containing products. This study revealed high levels of untreated caries with DMFT values of 1.225 and 0.65 for urban and rural children respectively (Table 1). This trend of high level of untreated caries parallels what was observed in several Sub-Saharan African countries [6, 14, 16–18]. This is an indicator of a general lack of professional oral health service in Zimbabwe. In addition, the high numbers of untreated teeth may also be a result of the low priority placed on oral health care compared with other needs. As previously described by Dye and Thornton-Evans [9] when people are faced with economic challenges they are unlikely to prioritize oral health unless they are well informed on the significance of maintaining good oral standards.

While previous studies in industrialized countries show a lower caries prevalence among children who reported prior consultation with a dentist [19],in this study; we observed a higher prevalence of dental caries among urban children who reported ever consulting a dentist (Table 2). We did not enquire on the reasons for consulting dentist by children in this study but it appears consultation was not for routine preventive clinical examination but likely for treatment of already existing problems. Routine annual dental check-ups that are common in industrialized countries are not affordable to most people in Zimbabwe; as a result children are only taken to a dentist as a last resort in most cases to have the teeth extracted. Previous studies [6, 16–18] have shown similar findings of untreated dental caries pointing to a common prevailing situation throughout Sub-Saharan Africa.

Despite limitations of this study which include use of a small convenient sample size drawn from only two schools coupled with our inability to verify the information reported on the questionnaire, our findings concur with previous studies done in Zimbabwe [7, 8] and several other Sub-Saharan Africa countries [6, 16–18]. Our data from the self-reported survey clearly shows deficits in healthy oral hygiene behavior while dental examinations revealed a high degree of inadequate dental care. Based on our findings, we advocate for a shift from the current restorative oriented dental services to preventative oriented services if an improvement in oral health delivery is to be achieved in Zimbabwe. Concerted effort from oral health professionals, schools, governments and the world health organization is required in order to increase general oral health awareness in both urban and rural areas. Evidence from developed countries shows that reduction of dental caries prevalence can only be achieved through targeted preventative and oral hygiene promotion programmes [19]. Since most urban children in this study reported not getting any information on oral health issues at school, one strategy that can used to increase oral health awareness among children is to incorporate oral health issues in the school curricula. We propose that schools could promote preventive oral health practices through various activities like educative dramas, poetry, music and art. In this study, no oral health education campaigns have reportedly been done in the two areas studied in a long time. Thus, the Ministry of Health in collaboration with the World Health Organization could also increase general oral health awareness by regularly holding national oral health awareness promotion campaigns. As a long term measure, it is important that Zimbabwe and other Sub-Saharan African countries support the training and education of oral health professionals who can develop and maintain oral health strategies and policies. We further recommend establishment of national systems for periodic collection, analysis and interpretation of oral health data for all indicator age groups.

## Conclusion

Our data shows a high prevalence of dental caries among 12 year old school children from low socio-economic status background in both urban and rural areas of Zimbabwe. This data may be of importance in the evaluation of the past and planning of future oral health prevention and treatment programs targeting young children in primary schools. A comprehensive community-focused oral health care intervention that includes oral health education in elementary schools and homes is recommended to increase general oral health awareness.

## References

[1] World health organization (2012). Oral health fact sheet N0 318.

[2] Petersen PE, Lennon MA (2004). Effective use of fluorides for the prevention of dental caries in the 21st century: the WHO approach. Community Dent Oral Epidemiol..

[3] Petersen PE, Bourgeois D, Ogawa H, Estupinan-Day S, Ndiaye C (2005). The Global burden of oral disease and risks to oral health. Bull World Health Organ..

[4] Kwan SYL, Petersen PE, Pine CM, Borutta A (2005). Health-promoting school; an opportunity for oral health promotion. Bulletin of the World Health organization..

[5] Slade GD (2001). Epidemiology of dental pain and dental caries among children and adolescents. Community Dent Health..

[6] Gathecha G, Makokha A, Wanzala P, Omolo J, Smith P (2012). Dental caries and oral health practices among 12 year old children in Nairobi West and Mathira West Districts, Kenya. Pan Afr Med J..

[7] Chironga L, Manji F (1989). Dental caries in 12-year-old urban and rural children in Zimbabwe. Community Dentistry and Oral Epidemiology..

[8] Frencken JE, Sithole WD, Mwaenga R, Htoon HM, Simon E (1999). National oral health survey Zimbabwe 1995: dental caries situation. Int Dent J..

[9] Dye BA, Thornton-Evans G (2010). Trends in oral health by poverty status as measured by Healthy People 2010 objectives. Public Health Rep..

[10] UNDP report on Zimbabwe. http://hdrstats.undp.org/en/countries/profiles/ZWE.html.

[11] Droz D, Gueguen R, Bruncher P, Gerhard JL, Roland E (2006). Epidemiological study of oral dental health of 4 year-old children in French nursery schools. Archives of Pediatrics and Adolescent Medicine..

[12] Moynihan PJ (2005). The role of diet and nutrition in the etiology and prevention of oral diseases. Bulletin of the World Health Organization..

[13] WHO: Oral health surveys (1997). Basic methods.

[14] Nurelhuda NM, Trovik TA, Ali RW, Ahmed MF (2009). Oral health status of 12-year-old school children in Khartoum state,the Sudan; a school-based survey. BMC Oral Health..

[15] Muwazi LM, Rwenyonyi CM, Tirwomwe FJ, Ssali C, Kasangaki A, Nkamba ME, Ekwaru P (2005). Prevalence of oral diseases/conditions in Uganda. Afr Health Sci..

[16] Mashoto KO, Åstrøm AN, David J, Masalu JR (2009). Dental pain, oral impacts and perceived need for dental treatment in Tanzanian school students: a cross-sectional study. Health and Quality of Life Outcomes..

[17] Bajomo AS, Rudolph MJ, Ogunbodede EO (2004). Dental caries in six, 12 and 15 year old Venda children in South Africa. East Afr Med J..

[18] Patel R (2012). The state of oral health in Europe. A report commissioned by the platform for better oral health in Europe.

